# Unraveling the functional instability of bacterial consortia in crude oil degradation via integrated co-occurrence networks

**DOI:** 10.3389/fmicb.2023.1270916

**Published:** 2023-10-12

**Authors:** Ping Li, Xiaolong Liang, Rongjiu Shi, Yongfeng Wang, Siqin Han, Ying Zhang

**Affiliations:** ^1^Key Laboratory of Pollution Ecology and Environmental Engineering, Institute of Applied Ecology, Chinese Academy of Sciences, Shenyang, China; ^2^University of Chinese Academy of Sciences, Beijing, China

**Keywords:** *Dietzia* genus, *Bacillus* genus, Co-occurrence networks, *Mycetocola* genus, functional potential, functional decline

## Abstract

**Introduction:**

Soil ecosystems are threatened by crude oil contamination, requiring effective microbial remediation. However, our understanding of the key microbial taxa within the community, their interactions impacting crude oil degradation, and the stability of microbial functionality in oil degradation remain limited.

**Methods:**

To better understand these key points, we enriched a crude oil-degrading bacterial consortium generation 1 (G1) from contaminated soil and conducted three successive transfer passages (G2, G3, and G4). Integrated Co-occurrence Networks method was used to analyze microbial species correlation with crude oil components across G1-G4.

**Results and discussion:**

In this study, G1 achieved a total petroleum hydrocarbon (TPH) degradation rate of 32.29% within 10 days. Through three successive transfer passages, G2-G4 consortia were established, resulting in a gradual decrease in TPH degradation to 23.14% at the same time. Specifically, saturated hydrocarbon degradation rates ranged from 18.32% to 14.17% among G1-G4, and only G1 exhibited significant aromatic hydrocarbon degradation (15.59%). Functional annotation based on PICRUSt2 and FAPROTAX showed that functional potential of hydrocarbons degradation diminished across generations. These results demonstrated the functional instability of the bacterial consortium in crude oil degradation. The relative abundance of the *Dietzia* genus showed the highest positive correlation with the degradation efficiency of TPH and saturated hydrocarbons (19.48, 18.38, *p* < 0.05, respectively), *Bacillus* genus demonstrated the highest positive correlation (21.94, *p* < 0.05) with the efficiency of aromatic hydrocarbon degradation. The key scores of *Dietzia* genus decreased in successive generations. A significant positive correlation (16.56, *p* < 0.05) was observed between the *Bacillus* and *Mycetocola* genera exclusively in the G1 generation. The decline in crude oil degradation function during transfers was closely related to changes in the relative abundance of key genera such as *Dietzia* and *Bacillus* as well as their interactions with other genera including *Mycetocola* genus. Our study identified key bacterial genera involved in crude oil remediation microbiome construction, providing a theoretical basis for the next step in the construction of the oil pollution remediation microbiome.

## 1. Introduction

Crude oil is a mixture of liquid hydrocarbons encompassing aliphatic, aromatics, resins, and asphaltenes (Varjani, 2017). When crude oil spills into the environment, it presents a potential hazard to both the ecosystem and biotic entities (Tang et al., 2011; Wang et al., 2021). Biological methods are more environment-friendly and cost-effective compared to physical and chemical methods (Vidonish et al., 2016; Johnson and Affam, 2018; Zhou et al., 2019). Microbial consortia are preferred over single microorganisms, because they possess distinct strengths in terms of degradation efficiency and adaptability to intricate environmental conditions (Abbasian et al., 2015; Gurav et al., 2017; Cui et al., 2020).

The constructing approaches of bacterial consortia include bottom-up and top-down (Lawson et al., 2019). Bottom-up is a random combination of single bacteria, which is also a common method to build crude oil degradation consortium. For example, several researchers constructed hydrocarbon–degradation consortia by combining biosurfactant–producing strains and crude oil–degrading strains (Lee et al., 2018; Chen et al., 2020; Cui et al., 2020; Dai et al., 2020), non–alkane–consuming and alkane degrader (Hu et al., 2020). The approach is full of randomness based on the complementary functions. By contrast, the top-down approach enriched specific function consortium by applying specific environmental pressure on the natural consortium (Lee et al., 2013). However, due to varying complexities of pollutants, functionality stability from top-down approach varies across successive generations. For microbial communities involved in degrading single-component contaminants, their degradation functionalities remain unaffected by the process of successive generations (Feng et al., 2019; Guo et al., 2021; Yin et al., 2021). However, for microbial communities engaged in degrading mixed pollutants, the stability of their degradation potential diminishes across generations (Chen et al., 2019; Gao et al., 2022; Lewin et al., 2022). Petroleum constitutes a complex mixture, adopting the top-down approach for selecting degradative microbial communities may lead to functional instability. To acquire microbial communities capable of crude oil degradation, it is vital to uncover the factors contributing to instability within top-down populations and identify crucial stabilizing species.

Keystone taxa were not numerically dominant in the communities (Banerjee et al., 2018), they had an effect on the structure and function of the consortium (Berry and Widder, 2014). By constructing species interaction networks, researchers have investigated microbial communities in groundwater and soil contaminated with hydrocarbons. They have identified key species exhibiting distinct traits: an elevated affinity for carbon sources, an abundance of genes related to hydrocarbon degradation (Ma et al., 2021), and the ability to produce metabolites that nourish other microorganisms (Geng et al., 2022; Jia et al., 2023). Recently, co–occurrence network analyses using high–throughput metagenomic data have been developed to identify key species and syntrophic relationships in microbial consortia. Involved measures include Pearson correlation, Spearman correlation, Bray–Curtis dissimilarity, Mutual information, and GBLM. Compared with Spearman and Pearson correlations, GBLM can mitigate spurious correlations among non–independent measurements as the increase in one relative abundance must be accompanied by a compositional decrease in another (Faust et al., 2012; Mac Aogain et al., 2021). Mac Aogain et al. (2021) integrated these five similarity measures to study disease-related microorganisms. To find key species and better understand the interrelationships within the microbial consortium, it would be beneficial to generate co–occurrence networks by merging the five similarity measures.

The objectives of this study were as follows: (1) enrich the crude oil–degrading bacterial consortia; (2) identify the key genera present and syntrophic relationships in the consortia; and (3) analyze the functional stability of the consortia through successive acclimation.

## 2. Materials and methods

### 2.1. Chemicals and reagents

Aerobic hydrocarbon degradation medium (AHDM), which was used for enriching crude oil–degrading bacterial populations, contains 10 g crude oil, 10 g (NH_4_)_2_SO_4_, 1.1 g KCl, 1.1 g NaCl, 1.97 g Na_2_HPO_4_, 0.22 g KH_2_PO_4_, 0.5 g MgSO_4_.7H_2_O, and 0.5 mL TES per liter. Trace element solution (TES) contains 0.56 g FeSO_4_, 0.17 g MnSO_4_, 0.25 g CuSO_4_, 0.24 g CaCl_2_, and 0.29 g ZnSO_4_, per liter of the solution. Petroleum was obtained from the Xinjiang oil field in China. All other chemicals and reagents used were of analytical grade.

### 2.2. Enrichment and successive transfer culture of crude oil–degrading bacterial consortia

Soil samples polluted with crude oil were collected near the Luliang oil field in Xinjiang, China. The soil samples showed that approximately 70% of the crude oil degraded after 2–3 months in a natural environment. To enrich the crude oil–degrading bacterial consortia, 5 g of soil was cultivated for 0.5 h at 200 rpm in a 100 mL flask containing 45 mL 0.9% NaCl under aerobic conditions, at 37°C, then allowed to stand for 1 h to obtain the leachate. The leachate was transferred to the shake flasks containing 100 mL AHDM and the culture was generation 1 (G1) culture. AHDM without any bacteria was used as the control. The AHDM was autoclaved at 121°C and 30 min before the cultivation experiment. The G1 cultures was incubated on an electrical shaker at 150 rpm and 37°C for 10d. At the end of cultivation, G1 culture was continuously transferred (10% v: v) into fresh 100 mL AHDM every 10 days. The subsequent culture generations were referred to by their consecutive batch culture numbers (G2, G3, and G4).

### 2.3. Separation of saturates, aromatics, resins, and asphaltenes

TPH content was assessed using a gravimetric method (Capelli et al., 2001). Briefly, residual oil from the culture (100 mL) was recovered by adding 40 mL carbon tetrachloride. The lower organic phase was filtered and dehydrated with anhydrous sodium sulfate. The TPH content was quantified gravimetrically, after solvent evaporation.

The residual oil was extracted and divided into saturates, aromatics, resins, and asphaltenes, based on the Chinese National Standard SY/T 5119–2016. Briefly, n-hexane was gradually added to the residual oil. The oil sample was left to stand for more than 12 h to fully precipitate the asphaltenes and measured. The filtrate was then subjected to chromatographic separation using a silica gel-neutral alumina column. Successive elution was performed using distinct organic solvents to sequentially recover the saturated hydrocarbon, aromatic hydrocarbon, and resin fractions. Saturated hydrocarbons were eluted with n-hexane, while a mixture of dichloromethane and n-hexane was employed for eluting aromatic hydrocarbons. Resin components were separated using anhydrous ethanol and chloroform as eluents. Following complete solvent evaporation, the weights of these three fractions were individually recorded.

### 2.4. DNA extraction and 16S rRNA gene-based community analysis

The medium was filtered through a sterile 0.45 μm organic membrane filter (Tianjin Jinteng experimental equipment Co. Ltd., Tianjin, China). Filtered membrane was utilized for DNA extraction. DNA extraction, PCR amplification, and sequencing were performed by Guangdong Magigene Biotechnology Co., Ltd. (Guangzhou, China). The V4 and V5 hypervariable regions of microbial 16S rRNA were amplified using the primers 515F (5′–GTGCCAGCMGCCG CGGTAA–3′) and 909R (5′–CCCCGYCAATTCMTTTRAGT–3′). The constructed libraries were pooled and sequenced using 250PE (paired–end) sequencing on an Illumina novaseq platform (Illumina, USA). Analysis of bacterial consortium composition and diversity was performed in triplicate. Demultiplexing, quality filtering, clustering into Amplicon-sequence variant (ASV), and construction of the ASV table were performed in QIIME2, v.2020.2 (Bolyen et al., 2019). The analysis yielded 1,503,255 high–quality reads (94.7% of reads averaged ≥ Q30 scores) distributed across 12 samples, with the minimum and maximum number of reads per sample being 88,513 and 171,446, respectively. The deblurring algorithm was used to construct the ASV table (Amir et al., 2017). The final ASV abundance table was then rarefied at 88513 sequences per sample through the alpha-rarefaction subcommand in QIIME2. Rarefaction curves based on observed ASV and the Good’s coverage index were used to evaluate the adequacy of the sampling depth, which was established at 88513 quality-filtered reads per sample. The feature classifier script implemented in QIIME2 was employed for the taxonomic assessment using the SILVA reference database, v. 138 (Quast et al., 2013). Alpha diversity was assessed using diversity (Shannon) and evenness indices. Beta diversity was assessed by computing the Bray–Curtis distance between the samples and by Principal coordinate analysis (PCoA). Core OTUs within G1-G4 were discerned based on specific criteria: OTUs with high frequency, present in over 80% of samples, and abundant OTUs with relative abundances exceeding 0.2% across the entirety of samples (Wu et al., 2019). The neighbor-joining phylogenetic tree of the 16S rRNA gene was constructed using MEGA11 software, employing representative sequences of highly abundant bacteria (relative abundances exceeding 1%). The resulting tree was then visualized using the Interactive Tree of Life (iTOL) platform.^1^ In this study, the potential functions of microbial consortia within G1-G4 were predicted by using Phylogenetic Investigation of Communities by Reconstruction of Unobserved States 2 (PICRUSt2, v2.5.2) and Functional Annotation of Prokaryotic Taxa (FAPROTAX, v1.2.4).

### 2.5. Co-occurrence analysis of microbial interaction

Weighted co–occurrence analysis with an ensemble of similarity measures (Bray–Curtis, Pearson, Spearman, and MI) and regression techniques (GBLM) (Faust et al., 2012) were used to generate microbial association networks (Mac Aogain et al., 2021).

Specifically, the input file containing the relative abundance of genera in G1–G4 was calculated by five diverse measures and obtained relationships (positive and negative) between genera in G1–G4. The Gephi and Cytoscape software was used to analyze Betweenness Centrality, Closeness Centrality, and Degree of nodes. Following the result of Banerjee et al. (2018), keystone species have the characteristics of low Betweenness Centrality, high Closeness Centrality, and high Degree. To obtain the scores of the genera of the keystone species, the three indices were homogenized and used Eq. 1:


(1) $$\begin{array}{l} \mathrm{Key}-\mathrm{scores}=\mathrm{Degree}+\mathrm{Closeness}\;\mathrm{Centrality}-\\ \;\mathrm{Betweeness}\;\mathrm{Centrality} \end{array}$$

### 2.6. Statistical analysis

An input file, which contained the relative abundance of all genera in G1–G4 and the degradation efficiency of TPH, saturates, and aromatics, calculated the relationship between the relative abundance of genera and the degradation efficiency of TPH, saturates, and aromatics by five diversity measures. Alpha diversity in different samples was compared using the Kruskal–Wallis pairwise test (Liddicoat et al., 2020). We used Permutational multivariate analysis of variance (PERMANOVA) to investigate the effects of time and four transfers on each component of beta diversity. Network metrics such as node degree, stress centrality, and betweenness centrality were calculated and visualized using Cytoscape. Statistical analysis and visualization of bacterial consortium and alpha and beta diversities were performed using the R packages reshape2, ggplot2, dplyr, VennDiagram, igraph, and vegan. The efficiency of crude oil degradation was assessed using Eq. 2


(2) $$\mathrm{Degradation}\;\mathrm{efficiency}=\frac{\mathrm{Residual}\;\mathrm{content}\;\mathrm{of}\;\left(\mathrm{control}-\mathrm{treat}\right)}{\mathrm{Residual}\;\mathrm{content}\;\mathrm{of}\;\mathrm{control}}$$

Data are expressed as mean ± standard deviation (SD) of three replicates. The Wilcoxon-Mann–Whitney test and Kruskal–Wallis test were performed for statistical analysis of values in different groups to determine the presence of significant differences.

## 3. Results

### 3.1. Crude oil biodegradation across four successive transfers

**Figure 1:**
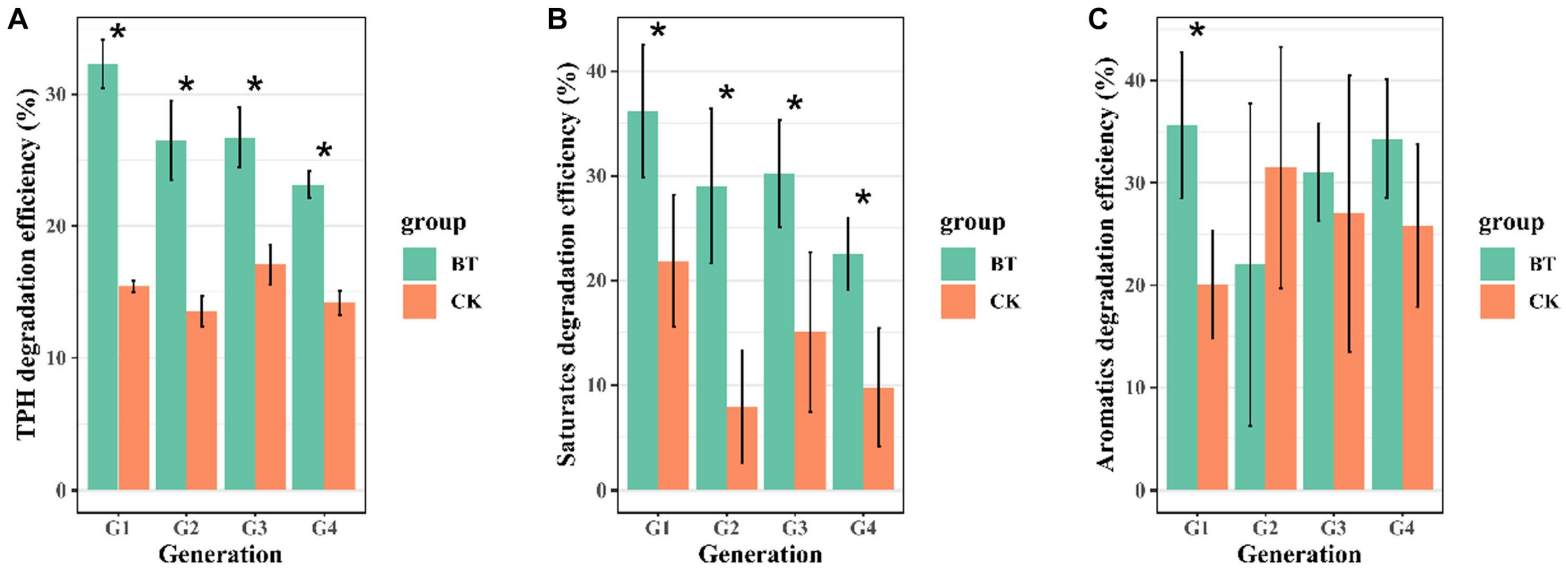
Degradation efficiency of crude oil and its two fractions in four successive transfers: **(A)** TPH degradation efficiency, **(B)** saturates degradation efficiency, and **(C)** aromatics degradation efficiency. The abbreviations used are CK for the control treatment and BT for the bacterial consortium treatment. The significance level is indicated by asterisks (*), with *p* < 0.05.

In this study, we enriched a crude oil-degrading bacterial consortium generation 1 (G1) from contaminated soil, achieving a TPH degradation rate of 32.29%. The crude oil degradation capacity dramatically decreased with G1 being continuously transferred. In comparison to G1, G4 consortium only achieved a crude oil degradation rate of 23.14% within the same incubation time (Figure 1A). The saturates hydrocarbons were degraded at 18.72, 22.89, 17.82, and 14.16% by bacterial consortia G1–G4 (Figure 1B), this indicates a decline in the degradation capacity of saturated hydrocarbons after G2. While the G1 consortium was capable of degrading aromatics, G2, G3, and G4 consortia exhibited negligible degradation of aromatics (Figure 1C), suggesting a loss in aromatics degradation capacity after G1. The degradation of resins and asphaltenes showed no significant difference between the bio-treatment and control groups (Supplementary Figures S1A,B). These results illustrate that TPH degradation efficiency declined during successive transfer cultures.

### 3.2. Dynamics of bacterial community composition

The rarefaction curve analysis indicated that the sequencing depth in our study was adequate to capture the diversity of bacterial communities in each sample, ensuring representative coverage (Supplementary Figure S2A). Furthermore, statistical analysis using the Kruskal-Wallis test revealed no significant differences in the Shannon diversity index and evenness index among the G1-G4 consortia (*p* > 0.1; Supplementary Figures S2B,C). When assessing beta diversity based on the Bray-Curtis distance, PERMANOVA analysis demonstrated no significant difference (*p* > 0.1) in bacterial community structure between G1-G4 consortia (Supplementary Figure S2D).

**Figure 2:**
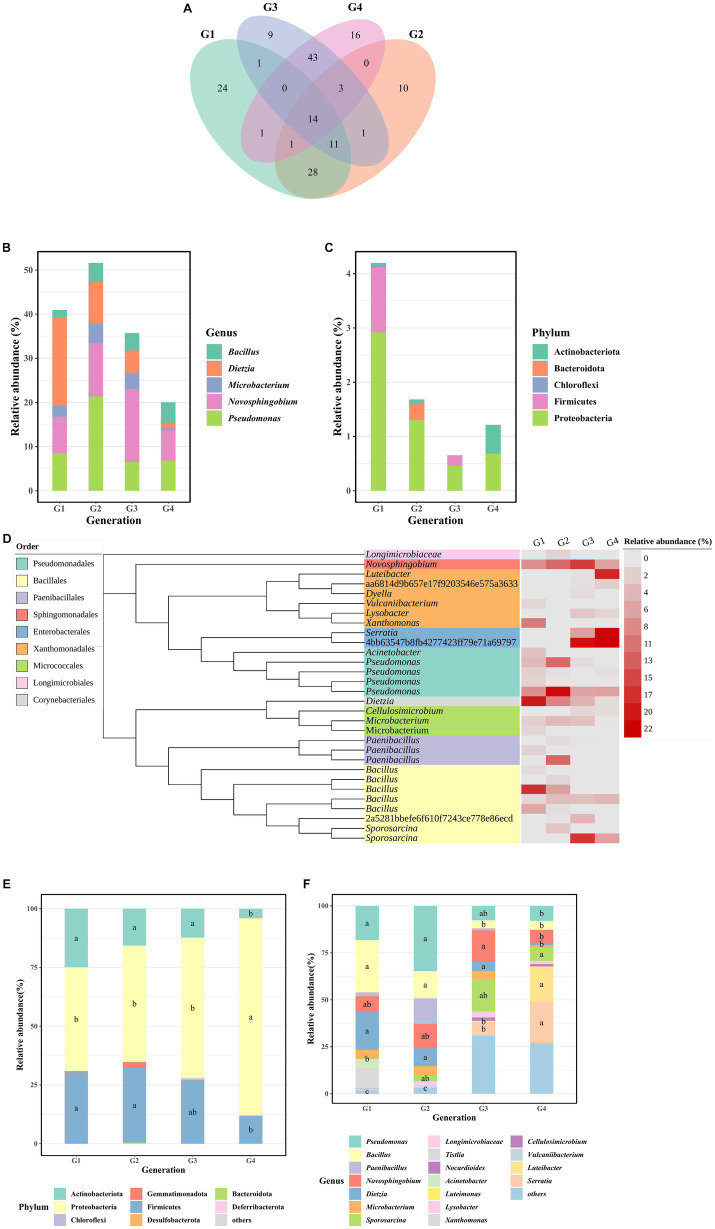
Bacterial taxonomic composition. **(A)** The Venn plot showing the shared and unique OTUs between G1–G4. **(B)** Core OTUs composition of four generations at the genus level. **(C)** Unique OTUs composition of four generations at the phylum level. **(D)** Taxonomic dendrogram showing the highly abundant bacteria of G1–G4. Color ranges identify order within the tree. Heatmap gradient represent the relative abundance of each OTU in G1-G4. **(E)** The relative abundance of bacterial phylum in the samples. **(F)** The relative abundance of the 10 most represent bacterial genera in the samples. Within each column, means with different letters are significantly different at *p* < 0.05.

Fourteen shared OTUs were observed in the four generations, while 24, 10, 9, and 16 unique OTUs were found, respectively, in G1, G2, G3, and G4 (Figure 2A). There were 5 OTUs in 14 shared OTUs identified as the regional core OTUs, accounting for 20.0 to 51.6% of the bacterial abundance of four generations (Figure 2B). All the 59 unique OTUs were classified to 5 phyla, including Actinobacteriota, Bacteroidota, Chloroflexi, Firmicutes, and Proteobacteria (Figure 2C). Compared to another generations, the unique OTUs of G1 were predominantly concentrated in Firmicutes and Proteobacteria. Those of G2 were mainly centered around Bacteroidota, and G4 primarily exhibited a concentration of Actinobacteriota.

In Figure 2D, the representative sequences of highly abundant bacteria (relative abundances exceeding 1%) in this study were selected to construct the phylogenetic tree. We found that the bacterial consortia of G1-G4 had the characteristic of high diversity. In G1, dominant OTUs were primarily concentrated in the *Bacillus*, *Dietzia*, *Novosphingobium*, *Xanthomonas* genus, and the Pseudomonadales order with *Acinetobacter* and *Pseudomonas* genera. In G2, predominant OTUs were mainly centered around *Pseudomonas*, *Dietzia*, *Novosphingobium*, and *Paenibacillus*. For G3, dominant OTUs were primarily found in *Novosphingobium*, *Sporosarcina* genus, and the Enterobacterales order. In G4, predominant OTUs were focused on the *Luteibacter* genus and the Enterobacterales order.

The analysis of the bacterial community revealed that Proteobacteria (59.35% ± 17.64%), Firmicutes (25.29% ± 9.18%), and Actinobacteriota (14.25% ± 8.63%) were the predominant phyla in G1-G4 consortia (Figure 2E). The dominant genera exhibited variations among the consortia. In G1, prevalent genera included *Pseudomonas* (18%), *Bacillus* (27.9%), *Dietzia* (20%), and *Novosphingobium* (8.27%). However, in G4, the dominant genera shifted to *Serratia* (22%), *Luteibacter* (18.9%), *Sporosarcina* (6.97%), *Bacillus* (4.77%), *Pseudomonas* (7.93%), and *Novosphingobium* (6.89%) (Figure 2F). Statistical analysis using the Wilcoxon-Mann–Whitney test indicated significant differences in the relative abundance of certain genera between G4 and the earlier generations. Specifically, the relative abundance of *Pseudomonas* in G4 was significantly lower than in G1 and G2, while *Bacillus* in G3 and G4 showed significantly lower abundance compared to G1 and G2. *Dietzia* in G4 exhibited a significantly lower abundance compared to G1-G3, and *Novosphingobium* in G4 showed a significantly lower abundance than in G3. By contrast, *Serratia* and *Luteibacter* in G4 had significantly higher abundance than in G3, and *Sporosarcina* in G4 showed significantly higher abundance than in G1 (Figure 2F).

### 3.3. Functional prediction analysis by FAPROTAX and PICRUSt2

**Figure 3:**
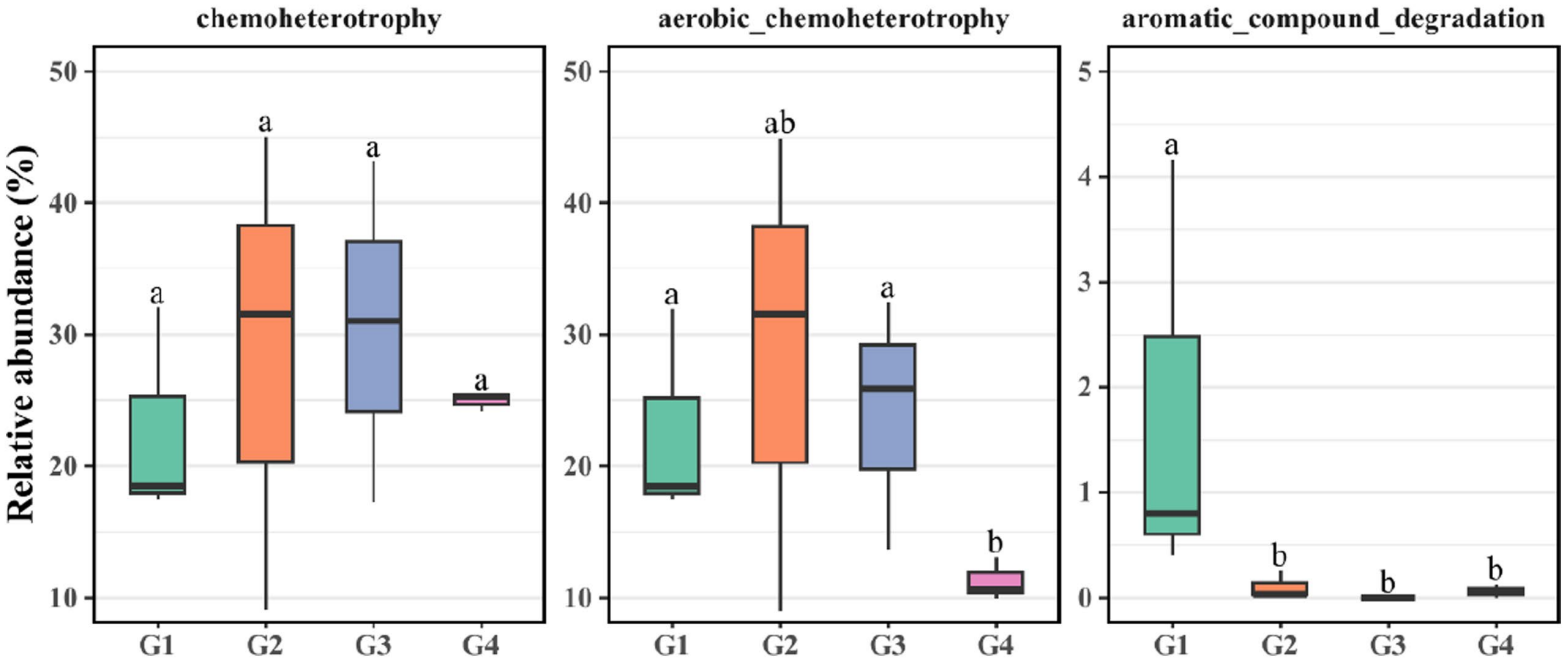
FAPROTAX analysis was utilized to evaluate the relative abundance of carbon transformation within G1–G4.

FAPROTAX and PICRUSt2 software were be used to predict the function of bacterial communities. FAPROTAX was used to predict the biochemical cycle of environmental samples. To display potential function of crude-oil degradation of bacterial communities, C transformation functions were promoted, including chemoheterotrophic, aerobic chemoheterotrophic, and aromatic_compound degradation processes (Figure 3). Our findings demonstrated that as successive generations progress, the aerobic chemoheterotrophic process of G1 and G3 became significantly more active in G4. The aromatic degradation processes of G1 exhibited pronounced activation in G2, G3, and G4. Notably, no significant differences in chemoorganotrophic were observed across G1 to G4.

**Figure 4:**
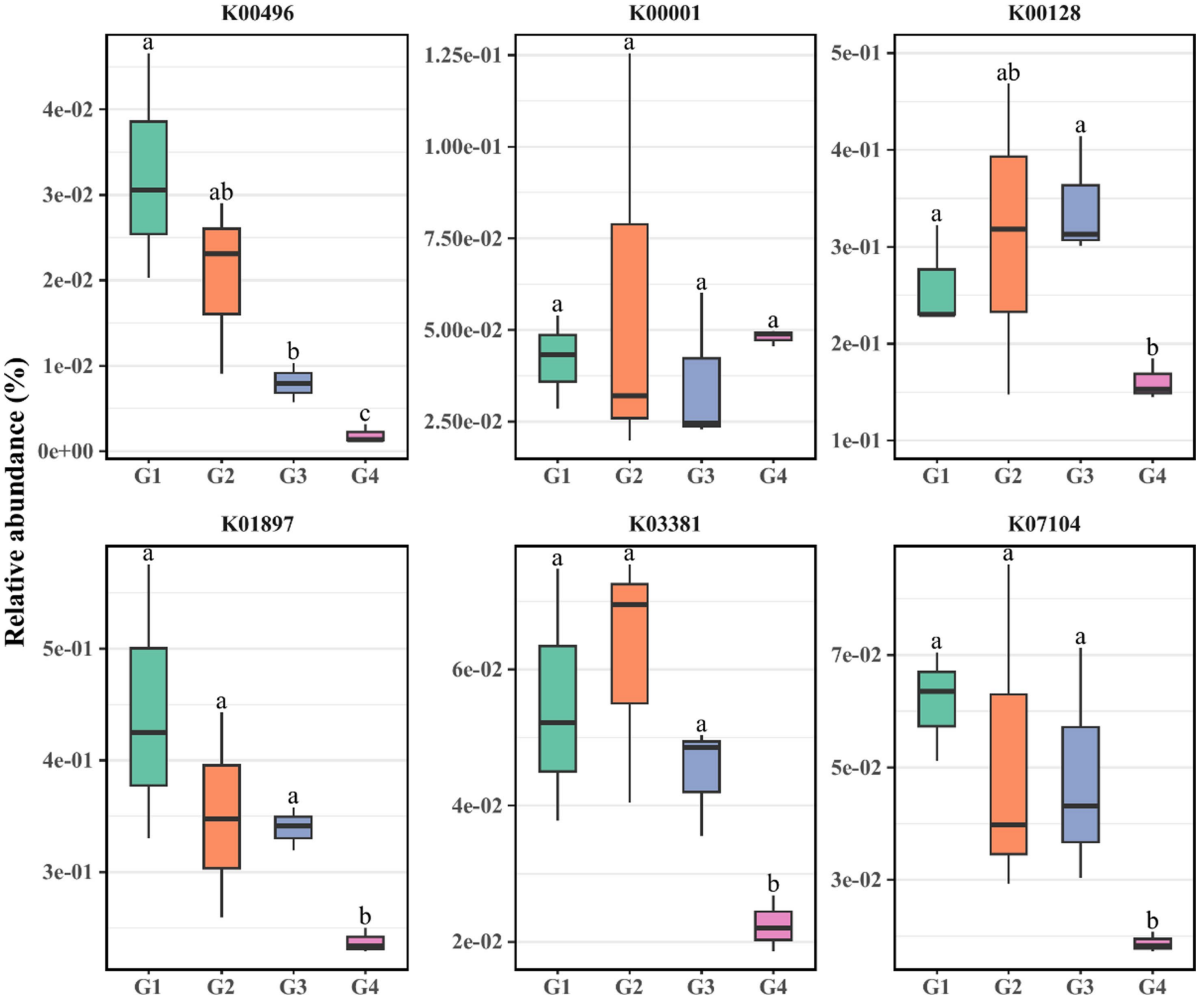
PICRUSt2 analysis was utilized to evaluate the relative abundance of hydrocarbon-degrading genes within G1–G4. K00496, *alkB*: alkane 1-monooxygenase [EC: 1.14.15.3]; K00001, *adh*: alcohol dehydrogenase [EC: 1.1.1.1]; K00128: aldehyde dehydrogenase (NAD+) [EC: 1.2.1.3]; K01897, *fadD*: long-chain acyl-CoA synthetase [EC: 6.2.1.3]; K03381, *catA*: catechol 1,2-dioxygenase [EC: 1.13.11.1]; K07104, *catE*: catechol 2,3-dioxygenase [EC: 1.13.11.2].

Alkanes primarily undergo intricate transformations facilitated by specific enzymatic systems such as alkane hydroxylases, ethanol dehydrogenases, acetaldehyde dehydrogenases, and acetyl-CoA. These processes culminate in their eventual entry into the fatty acid beta-oxidation pathway, leading to their complete oxidation into carbon dioxide and water (Rojo, 2009). The degradation of polycyclic aromatic hydrocarbons (PAHs) involves a series of sequential reactions including hydroxylation, dehydrogenation, isomerization, and ring cleavage. This intricate sequence results in the generation of intermediates that become integrated into the tricarboxylic acid (TCA) cycle. Subsequently, these intermediates undergo enzymatic conversions orchestrated by microorganisms, ultimately leading to the production of carbon dioxide and water. Notably, the ring cleavage process constitutes a pivotal step in the degradation of PAHs, involving two distinct pathways (Varjani, 2017): (1) ortho-cleavage catalyzed by catechol 1,2-dioxygenase (C12O) at ortho positions, and (2) meta-cleavage catalyzed by catechol 2,3-dioxygenase (C23O) at meta positions. Utilizing the PICRUSt2 software, we acquired the abundance of genes encoding these enzymes. As illustrated in the Figure 4, the relative abundance of the alkane hydroxylase gene (K00496) in G1 notably surpassed that in G3 and G4, while both G2 and G3 exceed G4. The acetaldehyde dehydrogenase gene (K00001) in G1 and G3 also exhibited significantly higher abundance than in G4. Furthermore, the acetyl-CoA gene (K01897), along with the C12O gene (K03381) and C23O gene (K07104) involved in aromatic hydrocarbon metabolism, exhibits significantly higher abundance in G1, G2, and G3 compared to G4. Notably, the ethanol dehydrogenase gene (K00128) displays negligible variation between G1 and G4. Consequently, the trend observed across G1 to G4 generations unveils a marked decline in the relative prevalence of genes linked to alkane and aromatic hydrocarbon metabolism, excluding the ethanol dehydrogenase gene.

### 3.4. Functional decline of bacterial consortia

**Figure 5:**
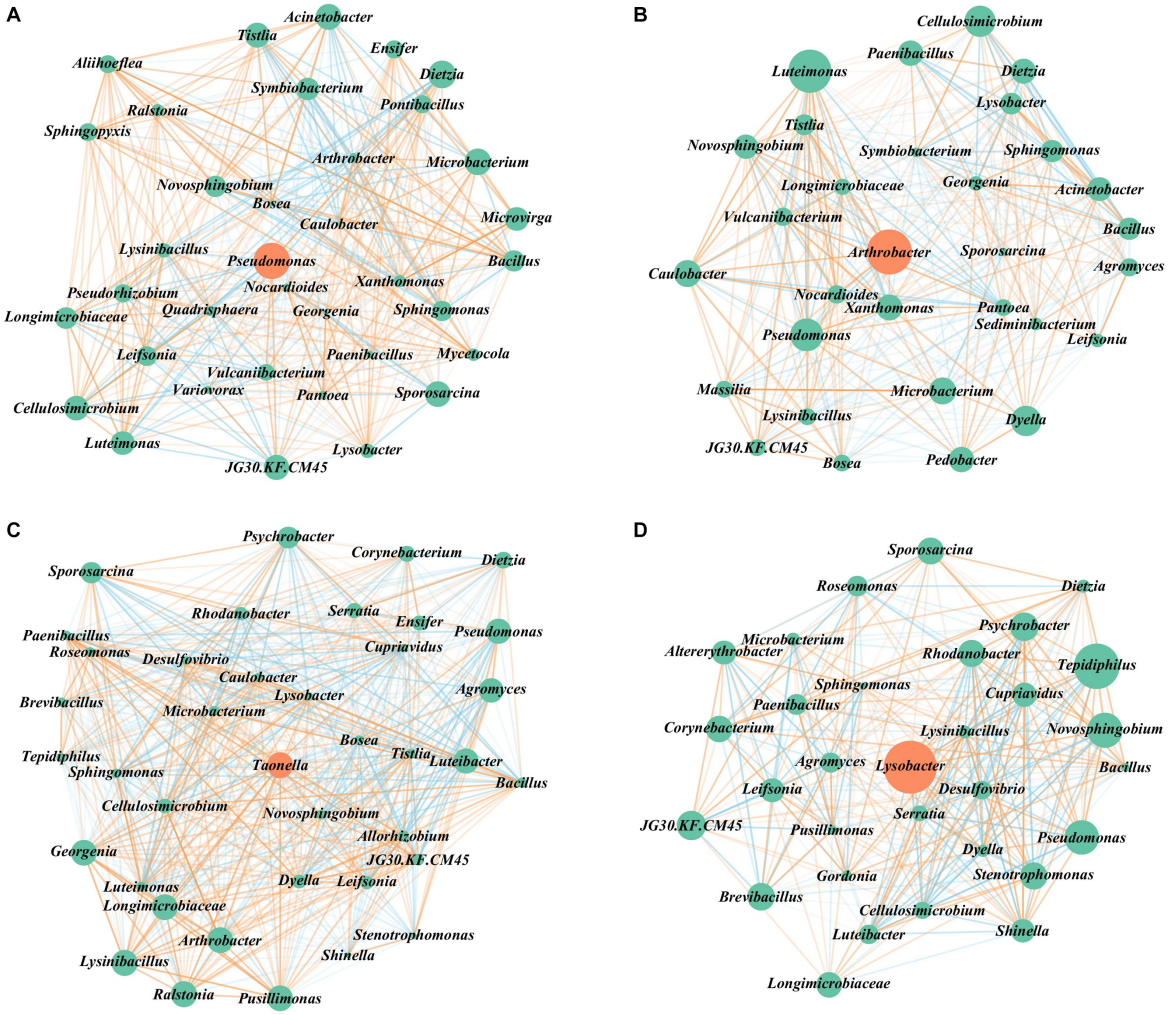
Co–occurrence analysis of bacterial network along successive transfer cultures. Interactions between microbes (nodes) are represented by connecting lines (edges), and the key–scores are reflected by node size. **(A–D)** Visualization of positive and negative interactions between all taxa on G1, G2, G3, G4. Interactions between microbes are classified as red if the sign of the edge weights between them is positive (positive correlation) and vice versa, the intensity of the colors reflected the strength of the correlations.

Based on key-score values and the relationship between genera, we constructed Figure 5. *Pseudomonas* exhibited the highest key-score in G1, followed by *Arthrobacter* in G2, *Taonella* in G3, and *Lysobacter* in G4 (Figures 5A–D). Their relative abundances were 18, 0.21, 0.03, and 1.77%, respectively (Supplementary Table S1). These findings indicate that the presence of key functional species in the consortium is not solely dependent on their relative abundance. Alternatively, important functional species can exist as either dominant populations with high relative abundance or non-dominant populations with low relative abundance.

Table 1 showed the correlation between the genus of top key scores in G1-G4 and the degradation efficiency of TPH, saturates, and aromatics. The correlations ranged from 15.42 to −9.54 for TPH, 11.91 to −9.79 for saturates, and 8.03 to −5.96 for aromatics. These results suggest a decline in the degradation capacity of TPH, saturated hydrocarbons, and aromatic hydrocarbons by the dominant species in the consortia. In Table 2, the highest positive correlation with TPH and saturated hydrocarbons degradation was observed for the *Dietzia* genus, with correlation values of 19.48 and 18.38, respectively (*p* < 0.05). Similarly, the *Bacillus* genus showed the highest positive correlation with aromatic hydrocarbons degradation, with a correlation value of 16.56 (*p* < 0.05). Conversely, in Table 3, *Serratia* exhibited the highest negative correlation with TPH and saturated hydrocarbon degradation, with correlation values of −19.38 and − 15.78, respectively (*p* < 0.05). Additionally, *Psychrobacter* showed the highest negative correlation with aromatic hydrocarbon degradation, with a correlation value of −8.92 (*p* < 0.05). These results emphasize the important roles of the *Dietzia* and *Bacillus* genera in the degradation of TPH, saturated hydrocarbons, and aromatic hydrocarbons, while highlighting the negative influence of *Serratia* and *Psychrobacter* on the degradation process.

**Table 1:** The relationship between the degradation of TPH, saturates, aromatics, and the genus of top key-scores.

Genus	The correlation coefficient with TPH degradation	The correlation coefficient with saturates degradation	The correlation coefficient with aromatics degradation
*Pseudomonas*	15.42*	11.91*	8.03*
*Arthrobacter*	4.02*	4.36*	3.10*
*Taonella*	−1.29*	−3.76*	−2.86*
*Lysobacter*	−9.54*	−9.79*	−5.96*

The significance level is indicated by asterisks (*), with *p* < 0.05.

**Table 2:** The strongest positive relationship between the degradation of TPH, saturates, aromatics, and the genera.

Genus	The correlation coefficient with TPH degradation	Genus	The correlation coefficient with saturates degradation	Genus	The correlation coefficient with aromatics degradation
*Dietzia*	19.48*	*Dietzia*	18.38*	*Bacillus*	21.94*
*Bacillus*	18.67*	*Bacillus*	14.50*	*Dietzia*	17.10*
*Pseudomonas*	15.42*	*Novosphingobium*	13.30*	*Xanthomonas*	16.93*
*Vulcaniibacterium*	12.41*	*Pseudomonas*	11.91*	*Pseudorhizobium*	14.42*
*Xanthomonas*	10.96*	*Vulcaniibacterium*	9.45*	*Acinetobacter*	12.63*
*Variovorax*	8.79*	*Variovorax*	9.35*	*Luteimonas*	12.25*
*Acinetobacter*	8.74*	*Luteimonas*	9.17*	*Vulcaniibacterium*	11.97*
*Luteimonas*	8.70*	*Symbiobacterium*	8.64*	*Mycetocola*	10.88*
*Pseudorhizobium*	7.78*	*Microbacterium*	6.91*	*Aliihoeflea*	10.84*
*Sphingomonas*	7.69*	*Tistlia*	6.24*	*Sphingopyxis*	10.84*

The significance level is indicated by asterisks (*), with *p* < 0.05.

**Table 3:** The strongest negative relationship between the degradation of TPH, saturates, aromatics, and the genera.

Genus	The correlation coefficient with TPH degradation	Genus	The correlation coefficient with saturates degradation	Genus	The correlation coefficient with aromatics degradation
*Serratia*	−19.38*	*Serratia*	−15.78*	*Psychrobacter*	−8.92*
*Luteibacter*	−16.42*	*Luteibacter*	−13.7*	*Corynebacterium*	−8.27*
*Novosphingobium*	−12.86*	*Microvirga*	−10.36*	*Serratia*	−8.2*
*Desulfovibrio*	−11.81*	*Altererythrobacter*	−10.16*	*Luteibacter*	−7.28*
*Altererythrobacter*	−11.08*	*Dyella*	−10.14*	*Agromyces*	−7.2*
*Psychrobacter*	−10.95*	*Desulfovibrio*	−10.09*	*Desulfovibrio*	−7.16*
*Gordonia*	−9.79*	*Lysobacter*	−9.79*	*Cellulosimicrobium*	−6.98*
*Lysobacter*	−9.54*	*Gordonia*	−9.66*	*Leifsonia*	−6.52*
*Cellulosimicrobium*	−9.35*	*Pusillimonas*	−9.14*	*Brevibacillus*	−6.37*
*Shinella*	−9.06*	*Psychrobacter*	−8.39*	*Tepidiphilus*	−6.19*

The significance level is indicated by asterisks (*), with *p* < 0.05.

The key scores of the *Dietzia* genus decreased from 3.62E-02 to 2.58E-02 during successive transfer cultures (Table 4). In contrast, the key scores of the *Serratia* and *Psychrobacter* genera increased from 0 to 2.87E-02 and 3.72E-02, respectively, after G2 (Table 4). The decline in the degradation of TPH and saturated hydrocarbons can be ascribed to the gradual weakening of the relationship between *Dietzia* and other genera in the consortia, as well as the progressive strengthening of the relationship between *Serratia* and *Psychrobacter* with other genera. These changes in inter-genera relationships within the consortia have significant implications for the overall degradation efficiency of TPH and saturated hydrocarbons.

**Table 4:** Betweenness Centrality, Closeness Centrality, Degree, and key–score of *Dietzia*, *Serratia*, and *Psychrobacter* at G1–G4.

Genus	Generation	Betweenness Centrality	Closeness Centrality	Degree	Key–score
*Dietzia*	G1	1.08E-02	2.49E-02	2.21E-02	3.62E-02
	G2	2.48E-02	3.05E-02	2.93E-02	3.50E-02
	G3	1.74E-02	2.35E-02	2.25E-02	2.86E-02
	G4	4.36E-02	3.39E-02	3.55E-02	2.58E-02
*Serratia*	G1	0	0	0	0
	G2	0	0	0	0
	G3	1.89E-02	2.35E-02	2.25E-02	2.71E-02
	G4	4.08E-02	3.39E-02	3.55–02	2.87E-02
*Psychrobacter*	G1	0	0	0	0
	G2	0	0	0	0
	G3	1.64E-02	2.45E-02	2.42E-02	3.23E-02
	G4	2.98E-02	3.30E-02	3.40E-02	3.72E-02

The *Bacillus* genus, exhibiting the highest positive correlation (16.56) with aromatic hydrocarbon degradation, exhibit positive correlation with *Mycetocola* genus only in G1 (Figures 5A–D). This correlation pattern aligns with the observed degradation pattern of aromatic hydrocarbons during successive transfer cultures, suggesting that the relationship between these two genera plays a role in influencing the degradation of aromatic hydrocarbons.

## 4. Discussion

In environmental bioremediation, microbial consortia are more effective in pollutant degradation compared to single microorganisms (Gurav et al., 2017). Unlike the stochastic nature of the bottom-up approach, the top-down method facilitates the acquisition of degradation microbial consortia with heightened environmental competitiveness. Nevertheless, microbial consortia enriched through the top-down approach necessitate multiple generations for practical application. In this study, we employed the top-down approach to cultivate a stable and efficient microbial consortium for crude oil degradation. However, degradation efficiency and related functional potential diminish across generations. To obtain more steadfast and competitively adept microbial consortia, a thorough understanding of instability reasons through microbial interactions becomes imperative. To explore microbial interactions, researchers often employ correlation analysis or build regression linear models to construct co-occurrence networks (Matchado et al., 2021). In order to obtain more reliable species relationships, a common approach is to integrate multiple analysis methods, which has been extensively applied in plant research (Duran et al., 2018; Zhang et al., 2018) and soil microbiome study (Mandakovic et al., 2018). However, when studying species relationships in petroleum-degrading microbial communities, current practices have been limited to utilizing a single method to construct co-occurrence networks (Alvarez-Barragan et al., 2022; Xiao et al., 2022; Zhou et al., 2023). To infer more robust species interactions in the investigation of petroleum-degrading microbial communities, we adopt a method similar to that proposed by Mac Aogain et al. (2021), integrating five different correlation analysis methods to build a co-occurrence network. This integration approach alleviated the occurrence of spurious correlations among species.

During successive transfers, the abundance of the most critical genera in each generation did not exhibit the highest values, contrary to the conventional belief that species importance within consortia is solely dictated by their relative abundance. In fact, rare microorganisms have been found to exert a significant positive impact on the degradation capacity of consortia, as demonstrated by Delgado-Baquerizo et al. (2016). The *Desulfosporosinus* genus, despite its scant representation (0.006%) among the total 16S rRNA genes in the microbial community of peatlands, exhibits exceptional efficiency in sulfate reduction (Pester et al., 2010). These findings underscore the crucial role of rare species in providing the necessary genetic resources for the intricate degradation processes that occur within consortia. The constrained proliferation of these rare species is likely attributed to specific environmental conditions that impose unfavorable growth circumstances, as proposed by Jousset et al. (2017).

The strongest positive correlation was observed between the degradation rates of total petroleum hydrocarbons (TPH) and saturated hydrocarbons and the abundance of the *Dietzia* genus. Numerous studies have reported the hydrocarbon degradation capacity of the *Dietzia* genus (Wang et al., 2011; Venil et al., 2021). Comparative genomic analysis has revealed that the genome of this genus contains a higher number of genes associated with lipid transport, metabolism, secondary metabolic production, synthesis, transport, and metabolism compared to other bacterial genomes (Fang et al., 2021). Lipid transport and metabolism play crucial roles in the degradation of components related to crude oil, while certain secondary metabolites, such as rhamnolipids, contribute to the emulsification of crude oil (Wang et al., 2014; Liu et al., 2018). Our study revealed an intriguing finding: the key scores of the *Dietzia* genus showed a gradual decline during successive transfers, indicating a weakening relationship between *Dietzia* and other genera in the consortium. However, when the *Dietzia* genus was co-cultured with other genera, it resulted in enhanced degradation of alkanes (Hu et al., 2020). This suggests that the declining relationship between *Dietzia* and other genera influences the efficiency of crude oil degradation. In conclusion, the relative abundance of the *Dietzia* genus in the consortia and its interaction with other genera play a significant role in the degradation of total oil and saturated hydrocarbons.

The highest positive correlation was observed between the degradation rate of aromatic hydrocarbons and the abundance of the *Bacillus* genus, which is well-known for its capability to degrade aromatic hydrocarbons (Das and Mukherjee, 2007; Eskandari et al., 2017; Ghorbannezhad et al., 2022). Interestingly, our analysis revealed a significant positive correlation between the *Bacillus* genus and the *Mycetocola* genus, but only in the G1 generation of the bacterial consortia. This correlation aligns with the observed pattern of aromatic hydrocarbon degradation during successive transfers. *Mycetocola* genus has been reported to counteract the toxic effects of tolaasin I produced by the *Pseudomonas* genus, which inhibits the growth of both Gram-negative bacteria like *Escherichia coli* and Gram-positive bacteria like *Bacillus subtilis* (Rainey et al., 1991; Hermenau et al., 2020; Castaldi et al., 2022). Notably, in the G1-G4 bacterial consortia, the *Pseudomonas* genus was consistently present with a relative abundance above 7.93%. Therefore, we speculate that the lack of *Mycetocola* genus in the G2–G4 consortia could have resulted in the persistence of the toxic effect of tolaasin I, thereby limiting the degradation of aromatic hydrocarbons by the *Bacillus* genus. In summary, our findings suggest that the abundance of the *Bacillus* genus is positively correlated with the degradation of aromatic hydrocarbons. Furthermore, the presence of the *Mycetocola* genus appears to be crucial in alleviating the toxic effects of tolaasin I and facilitating the degradation of aromatic hydrocarbons by the *Bacillus* genus.

During successive transfers, we observed that the degradation rates of total petroleum hydrocarbons (TPH) and saturated hydrocarbons exhibited the strongest negative correlation with the relative abundance of the *Serratia* genus. Similarly, the degradation rates of aromatic hydrocarbons showed the highest negative correlation with the relative abundance of the *Psychrobacter* genus. It is noteworthy that both the *Serratia* and *Psychrobacter* genera have been reported to possess crude oil degradation capabilities (Fagbemi and Sanusi, 2017; Lasek et al., 2017; Semai et al., 2021). By analyzing the relationships between the *Serratia* and *Psychrobacter* genera and other bacterial species, we noticed a gradual increase in the key scores of these genera within the bacterial consortia during successive transfers. This suggests a strengthening association between these genera and other members of the consortium. Considering the degradation capabilities of *Serratia* and *Psychrobacter* genera, it is reasonable to speculate that the competition among microbial species within the consortium contributed to a decline in the overall crude oil degradation capacity (Abtahi et al., 2020).

By employing network analysis methods, we can provide insights into the underlying causes of the functional decline in crude oil degradation. Nonetheless, to substantiate our research outcomes, it is imperative to conduct subsequent experimental studies focusing on the pertinent microorganisms and genes. These additional investigations will contribute to a more comprehensive and robust understanding of the complex interactions within the microbial community and their implications for crude oil degradation.

## 5. Conclusion

The relevant functional potential and degradation capacity of the bacterial consortium for crude oil gradually declined during the successive transfers. To investigate the factors influencing the degradation function of the bacterial consortia, we employed the 16S rRNA amplification technique and conducted bioinformatics statistical analysis. Our results revealed that the relative abundance of key genera within the bacterial consortia was not the sole determinant of their importance. The relative abundance of the *Dietzia* genus and its interactions with other genera emerged as critical factors influencing the degradation of TPH and saturated hydrocarbons. Furthermore, the decreasing relative abundance of the *Bacillus* genus and its interaction with the *Mycetocola* genus were found to impact the degradation of aromatic hydrocarbons. These findings highlight the intricate and dynamic nature of microbial interactions within crude oil degradation processes, underscoring the importance of gaining a comprehensive understanding of microbial community dynamics for the development of effective bioremediation strategies.

## Data availability statement

The datasets presented in this study can be found in online repositories. The names of the repository/repositories and accession number (s) can be found at: https://www.ncbi.nlm.nih.gov/, PRJNA997000.

## Author contributions

PL: Conceptualization, Methodology, Writing – original draft. XL: Writing – original draft. RS: Conceptualization, Writing – review & editing. YW: Methodology, Writing – review & editing. SH: Methodology, Writing – review & editing. YZ: Conceptualization, Writing – original draft, Writing – review & editing.

## Footnotes

^1^https://itol.embl.de/

## References

[ref1] Abbasian F. Lockington R. Mallavarapu M. Naidu R. (2015). A comprehensive review of aliphatic hydrocarbon biodegradation by bacteria. Appl. Biochem. Biotechnol. 176, 670–699. doi: 10.1007/s12010-015-1603-5, PMID: 25935219

[ref2] Abtahi H. Parhamfar M. Saeedi R. Villasenor J. Sartaj M. Kumar V.. (2020). Effect of competition between petroleum-degrading bacteria and indigenous compost microorganisms on the efficiency of petroleum sludge bioremediation: field application of mineral-based culture in the composting process. J. Environ. Manag. 258:110013. doi: 10.1016/j.jenvman.2019.110013, PMID: 31929055

[ref3] Alvarez-Barragan J. Cravo-Laureau C. Duran R. (2022). Fungal-bacterial network in PAH-contaminated coastal marine sediment. Environ. Sci. Pollut. Res. Int. 29, 72718–72728. doi: 10.1007/s11356-022-21012-4, PMID: 35614354

[ref4] Amir A. McDonald D. Navas-Molina J. A. Kopylova E. Morton J. T. Zech Xu Z.. (2017). Deblur rapidly resolves single-nucleotide community sequence patterns. mSystems 2:e00191-16. doi: 10.1128/mSystems.00191-16, PMID: 28289731 PMC5340863

[ref5] Banerjee S. Schlaeppi K. van der Heijden M. G. A. (2018). Keystone taxa as drivers of microbiome structure and functioning. Nat. Rev. Microbiol. 16, 567–576. doi: 10.1038/s41579-018-0024-1, PMID: 29789680

[ref6] Berry D. Widder S. (2014). Deciphering microbial interactions and detecting keystone species with co-occurrence networks. Front. Microbiol. 5:219. doi: 10.3389/fmicb.2014.00219, PMID: 24904535 PMC4033041

[ref7] Bolyen E. Rideout J. R. Dillon M. R. Bokulich N. A. Abnet C. C. Al-Ghalith G. A.. (2019). Reproducible, interactive, scalable and extensible microbiome data science using QIIME 2. Nat. Biotechnol. 37, 852–857. doi: 10.1038/s41587-019-0209-9, PMID: 31341288 PMC7015180

[ref8] Capelli S. M. Busalmen J. P. De Sanchez S. R. (2001). Hydrocarbon bioremediation of a mineral-base contaminated waste from crude oil extraction by indigenous bacteria. Int. Biodeterior. Biodegradation 47, 233–238. doi: 10.1016/S0964-8305(01)00050-6

[ref9] Castaldi S. Cimmino A. Masi M. Evidente A. (2022). Bacterial lipodepsipeptides and some of their derivatives and cyclic dipeptides as potential agents for biocontrol of pathogenic bacteria and fungi of agrarian plants. J. Agric. Food Chem. 70, 4591–4598. doi: 10.1021/acs.jafc.1c08139, PMID: 35395154 PMC9026286

[ref10] Chen W. Kong Y. Li J. Sun Y. Min J. Hu X. (2020). Enhanced biodegradation of crude oil by constructed bacterial consortium comprising salt-tolerant petroleum degraders and biosurfactant producers. Int. Biodeterior. Biodegradation 154:105047. doi: 10.1016/j.ibiod.2020.105047

[ref11] Chen Y. Wang C. Dong S. Jiang L. Shi Y. Li X.. (2019). Microbial community assembly in detergent wastewater treatment bioreactors: influent rather than inoculum source plays a more important role. Bioresour. Technol. 287:121467. doi: 10.1016/j.biortech.2019.121467, PMID: 31121447

[ref12] Cui J. Huang L. Wang W. Xu P. Zanaroli G. Tang H. (2020). Maximization of the petroleum biodegradation using a synthetic bacterial consortium based on minimal value algorithm. Int. Biodeterior. Biodegradation 150:104964. doi: 10.1016/j.ibiod.2020.104964

[ref13] Dai X. Lv J. Guo S. Wei W. (2020). Heavy oil biodegradation by mixed bacterial consortium of biosurfactant-producing and heavy oil-degrading Bacteria. Pol. J. Environ. Stud. 30, 71–80. doi: 10.15244/pjoes/120769

[ref14] Das K. Mukherjee A. K. (2007). Crude petroleum-oil biodegradation efficiency of *Bacillus subtilis* and *Pseudomonas aeruginosa* strains isolated from a petroleum-oil contaminated soil from North-East India. Bioresour. Technol. 98, 1339–1345. doi: 10.1016/j.biortech.2006.05.032 16828284

[ref15] Delgado-Baquerizo M. Giaramida L. Reich P. B. Khachane A. N. Hamonts K. Edwards C.. (2016). Lack of functional redundancy in the relationship between microbial diversity and ecosystem functioning. J. Ecol. 104, 936–946. doi: 10.1111/1365-2745.12585

[ref16] Duran P. Thiergart T. Garrido-Oter R. Agler M. Kemen E. Schulze-Lefert P.. (2018). Microbial interkingdom interactions in roots promote *Arabidopsis* survival. Cells 175, 973–983.e14. doi: 10.1016/j.cell.2018.10.020, PMID: 30388454 PMC6218654

[ref17] Eskandari S. Hoodaji M. Tahmourespour A. Abdollahi A. Baghi T. Eslamian S.. (2017). Bioremediation of polycyclic aromatic hydrocarbons by *Bacillus Licheniformis* ATHE9 and *Bacillus Mojavensis* ATHE13 as newly strains isolated from oil-contaminated soil. J. Geogr. Environ. Earth Sci. Int. 11, 1–11. doi: 10.9734/jgeesi/2017/35447

[ref18] Fagbemi O. K. Sanusi A. I. (2017). Chromosomal and plasmid mediated degradation of crude oil by *Bacillus coagulans*, *Citrobacter koseri* and *Serratia ficaria* isolated from the soil. Afr. J. Biotechnol. 16, 1242–1253. doi: 10.5897/ajb2017.15960

[ref19] Fang H. Xu J. B. Nie Y. Wu X. L. (2021). Pan-genomic analysis reveals that the evolution of *Dietzia* species depends on their living habitats. Environ. Microbiol. 23, 861–877. doi: 10.1111/1462-2920.15176, PMID: 32715552

[ref20] Faust K. Sathirapongsasuti J. F. Izard J. Segata N. Gevers D. Raes J.. (2012). Microbial co-occurrence relationships in the human microbiome. PLoS Comput. Biol. 8:e1002606. doi: 10.1371/journal.pcbi.1002606, PMID: 22807668 PMC3395616

[ref21] Feng N. X. Yu J. Xiang L. Yu L. Y. Zhao H. M. Mo C. H.. (2019). Co-metabolic degradation of the antibiotic ciprofloxacin by the enriched bacterial consortium XG and its bacterial community composition. Sci. Total Environ. 665, 41–51. doi: 10.1016/j.scitotenv.2019.01.322, PMID: 30772572

[ref22] Gao J. Qin J. Ye F. Ding F. Liu G. Li A.. (2022). Constructing simplified microbial consortia to improve the key flavour compounds during strong aroma-type Baijiu fermentation. Int. J. Food Microbiol. 369:109594. doi: 10.1016/j.ijfoodmicro.2022.109594, PMID: 35299048

[ref23] Geng S. Xu G. You Y. Xia M. Zhu Y. Ding A.. (2022). Occurrence of polycyclic aromatic compounds and interdomain microbial communities in oilfield soils. Environ. Res. 212:113191. doi: 10.1016/j.envres.2022.113191, PMID: 35351456

[ref24] Ghorbannezhad H. Moghimi H. Dastgheib S. M. M. (2022). Biodegradation of high molecular weight hydrocarbons under saline condition by halotolerant *Bacillus subtilis* and its mixed cultures with Pseudomonas species. Sci. Rep. 12:13227. doi: 10.1038/s41598-022-17001-9, PMID: 35918482 PMC9345985

[ref25] Guo Z. Yin H. Wei X. Zhu M. Lu G. Dang Z. (2021). Effects of methanol on the performance of a novel BDE-47 degrading bacterial consortium QY2 in the co-metabolism process. J. Hazard. Mater. 415:125698. doi: 10.1016/j.jhazmat.2021.125698, PMID: 33773249

[ref26] Gurav R. Lyu H. Ma J. Tang J. Liu Q. Zhang H. (2017). Degradation of n-alkanes and PAHs from the heavy crude oil using salt-tolerant bacterial consortia and analysis of their catabolic genes. Environ. Sci. Pollut. Res. Int. 24, 11392–11403. doi: 10.1007/s11356-017-8446-2 28315056

[ref27] Hermenau R. Kugel S. Komor A. J. Hertweck C. (2020). Helper bacteria halt and disarm mushroom pathogens by linearizing structurally diverse cyclolipopeptides. Proc. Natl. Acad. Sci. U. S. A. 117, 23802–23806. doi: 10.1073/pnas.2006109117, PMID: 32868430 PMC7519232

[ref28] Hu B. Wang M. Geng S. Wen L. Wu M. Nie Y.. (2020). Metabolic exchange with non-alkane-consuming *Pseudomonas stutzeri* SLG510A3-8 improves n-alkane biodegradation by the alkane degrader *Dietzia* sp. strain DQ12-45-1b. Appl. Environ. Microbiol. 86, e02931–e02919. doi: 10.1128/AEM.02931-19, PMID: 32033953 PMC7117941

[ref29] Jia W. Cheng L. Tan Q. Liu Y. Dou J. Yang K.. (2023). Response of the soil microbial community to petroleum hydrocarbon stress shows a threshold effect: research on aged realistic contaminated fields. Front. Microbiol. 14:1188229. doi: 10.3389/fmicb.2023.1188229, PMID: 37389339 PMC10301742

[ref30] Johnson O. A. Affam A. C. (2018). Petroleum sludge treatment and disposal: a review. Environ. Eng. Res. 24, 191–201. doi: 10.4491/eer.2018.134

[ref31] Jousset A. Bienhold C. Chatzinotas A. Gallien L. Gobet A. Kurm V.. (2017). Where less may be more: how the rare biosphere pulls ecosystems strings. ISME J. 11, 853–862. doi: 10.1038/ismej.2016.174, PMID: 28072420 PMC5364357

[ref32] Lasek R. Dziewit L. Ciok A. Decewicz P. Romaniuk K. Jedrys Z.. (2017). Genome content, metabolic pathways and biotechnological potential of the psychrophilic Arctic bacterium *Psychrobacter* sp. DAB_AL43B, a source and a host of novel *Psychrobacter*-specific vectors. J. Biotechnol. 263, 64–74. doi: 10.1016/j.jbiotec.2017.09.011, PMID: 28919459

[ref33] Lawson C. E. Harcombe W. R. Hatzenpichler R. Lindemann S. R. Löffler F. E. O'Malley M. A.. (2019). Common principles and best practices for engineering microbiomes. Nat. Rev. Microbiol. 17, 725–741. doi: 10.1038/s41579-019-0255-9, PMID: 31548653 PMC8323346

[ref34] Lee Y. Jeong S. E. Hur M. Ko S. Jeon C. O. (2018). Construction and evaluation of a Korean native microbial consortium for the bioremediation of diesel fuel-contaminated soil in Korea. Front. Microbiol. 9:2594. doi: 10.3389/fmicb.2018.02594, PMID: 30425703 PMC6218622

[ref35] Lee D. J. Show K. Y. Wang A. (2013). Unconventional approaches to isolation and enrichment of functional microbial consortium--a review. Bioresour. Technol. 136, 697–706. doi: 10.1016/j.biortech.2013.02.075, PMID: 23566469

[ref36] Lewin G. R. Davis N. M. McDonald B. R. Book A. J. Chevrette M. G. Suh S.. (2022). Long-term cellulose enrichment selects for highly cellulolytic consortia and competition for public goods. Msystems 7, e0151921–e0101521. doi: 10.1128/msystems.01519-21, PMID: 35258341 PMC9040578

[ref37] Liddicoat C. Sydnor H. Cando-Dumancela C. Dresken R. Liu J. Gellie N. J. C.. (2020). Naturally-diverse airborne environmental microbial exposures modulate the gut microbiome and may provide anxiolytic benefits in mice. Sci. Total Environ. 701:134684. doi: 10.1016/j.scitotenv.2019.134684 31704402

[ref38] Liu G. Zhong H. Yang X. Liu Y. Shao B. Liu Z. (2018). Advances in applications of rhamnolipids biosurfactant in environmental remediation: a review. Biotechnol. Bioeng. 115, 796–814. doi: 10.1002/bit.26517, PMID: 29240227

[ref39] Ma Y. Zhao H. Shan Q. Xu Y. Yu M. Cui J.. (2021). K-strategy species plays a pivotal role in the natural attenuation of petroleum hydrocarbon pollution in aquifers. J. Hazard. Mater. 420:126559. doi: 10.1016/j.jhazmat.2021.126559, PMID: 34252660

[ref40] Mac Aogain M. Narayana J. K. Tiew P. Y. Ali N. Yong V. F. L. Jaggi T. K.. (2021). Integrative microbiomics in bronchiectasis exacerbations. Nat. Med. 27, 688–699. doi: 10.1038/s41591-021-01289-7, PMID: 33820995

[ref41] Mandakovic D. Rojas C. Maldonado J. Latorre M. Travisany D. Delage E.. (2018). Structure and co-occurrence patterns in microbial communities under acute environmental stress reveal ecological factors fostering resilience. Sci. Rep. 8:5875. doi: 10.1038/s41598-018-23931-0, PMID: 29651160 PMC5897386

[ref42] Matchado M. S. Lauber M. Reitmeier S. Kacprowski T. Baumbach J. Haller D.. (2021). Network analysis methods for studying microbial communities: a mini review. Comput. Struct. Biotechnol. J. 19, 2687–2698. doi: 10.1016/j.csbj.2021.05.001, PMID: 34093985 PMC8131268

[ref43] Pester M. Bittner N. Deevong P. Wagner M. Loy A. (2010). A 'rare biosphere' microorganism contributes to sulfate reduction in a peatland. ISME J. 4, 1591–1602. doi: 10.1038/ismej.2010.75, PMID: 20535221 PMC4499578

[ref44] Quast C. Pruesse E. Yilmaz P. Gerken J. Schweer T. Yarza P.. (2013). The SILVA ribosomal RNA gene database project: improved data processing and web-based tools. Nucleic Acids Res. 41, D590–D596. doi: 10.1093/nar/gks1219, PMID: 23193283 PMC3531112

[ref45] Rainey P. B. Brodey C. L. Johnstone K. (1991). Biological properties and spectrum of activity of tolaasin, a lipodepsipeptide toxin produced by the mushroom pathogen *Pseudomonas tolaasii*. Physiol. Mol. Plant Pathol. 39, 57–70. doi: 10.1016/0885-5765(91)90031-C

[ref46] Rojo F. (2009). Degradation of alkanes by bacteria. Environ. Microbiol. 11, 2477–2490. doi: 10.1111/j.1462-2920.2009.01948.x 19807712

[ref47] Semai A. Plewniak F. Charrié-Duhaut A. Sayeh A. Gil L. Vandecasteele C.. (2021). Characterisation of hydrocarbon degradation, biosurfactant production, and biofilm formation in *Serratia* sp. Tan 611: a new strain isolated from industrially contaminated environment in Algeria. Antonie Van Leeuwenhoek 114, 411–424. doi: 10.1007/s10482-021-01527-5, PMID: 33587226

[ref48] Tang J. Wang M. Wang F. Sun Q. Zhou Q. (2011). Eco-toxicity of petroleum hydrocarbon contaminated soil. J. Environ. Sci. 23, 845–851. doi: 10.1016/s1001-0742(10)60517-7, PMID: 21790059

[ref49] Varjani S. J. (2017). Microbial degradation of petroleum hydrocarbons. Bioresour. Technol. 223, 277–286. doi: 10.1016/j.biortech.2016.10.037 27789112

[ref50] Venil C. K. Malathi M. Devi P. R. (2021). Characterization of *Dietzia maris* AURCCBT01 from oil-contaminated soil for biodegradation of crude oil. 3 Biotech 11:291. doi: 10.1007/s13205-021-02807-7, PMID: 34109094 PMC8141481

[ref51] Vidonish J. E. Zygourakis K. Masiello C. A. Sabadell G. Alvarez P. J. J. (2016). Thermal treatment of hydrocarbon-impacted soils: a review of technology innovation for sustainable remediation. Engineering 2, 426–437. doi: 10.1016/j.eng.2016.04.005

[ref52] Wang W. Cai B. Shao Z. (2014). Oil degradation and biosurfactant production by the deep sea bacterium *Dietzia maris* As-13-3. Front. Microbiol. 5:711. doi: 10.3389/fmicb.2014.00711, PMID: 25566224 PMC4267283

[ref53] Wang X. B. Chi C. Q. Nie Y. Tang Y. Q. Tan Y. Wu G.. (2011). Degradation of petroleum hydrocarbons (C6-C40) and crude oil by a novel *Dietzia* strain. Bioresour. Technol. 102, 7755–7761. doi: 10.1016/j.biortech.2011.06.009, PMID: 21715162

[ref54] Wang S. Wang D. Yu Z. Dong X. Liu S. Cui H.. (2021). Advances in research on petroleum biodegradability in soil. Environ. Sci.: Processes Impacts 23, 9–27. doi: 10.1039/d0em00370k, PMID: 33393551

[ref55] Wu L. Ning D. Zhang B. Li Y. Zhang P. Shan X.. (2019). Global diversity and biogeography of bacterial communities in wastewater treatment plants. Nat. Microbiol. 4, 1183–1195. doi: 10.1038/s41564-019-0426-5, PMID: 31086312

[ref56] Xiao X. Zheng Q. Shen R. Huang K. Xu H. Tu B.. (2022). Patterns of groundwater bacterial communities along the petroleum hydrocarbon gradient. J. Environ. Chem. Eng. 10:108773. doi: 10.1016/j.jece.2022.108773

[ref57] Yin T. Lin H. Dong Y. Li B. He Y. Liu C.. (2021). A novel constructed carbonate-mineralized functional bacterial consortium for high-efficiency cadmium biomineralization. J. Hazard. Mater. 401:123269. doi: 10.1016/j.jhazmat.2020.123269 32623308

[ref58] Zhang B. Zhang J. Liu Y. Shi P. Wei G. (2018). Co-occurrence patterns of soybean rhizosphere microbiome at a continental scale. Soil Biol. Biochem. 118, 178–186. doi: 10.1016/j.soilbio.2017.12.011

[ref59] Zhou H. Liu Q. Jiang L. Shen Q. Chen C. Zhang C.. (2023). Enhanced remediation of oil-contaminated intertidal sediment by bacterial consortium of petroleum degraders and biosurfactant producers. Chemosphere 330:138763. doi: 10.1016/j.chemosphere.2023.138763, PMID: 37094722

[ref60] Zhou Z. Liu X. Sun K. Lin C. Ma J. He M.. (2019). Persulfate-based advanced oxidation processes (AOPs) for organic-contaminated soil remediation: a review. Chem. Eng. J. 372, 836–851. doi: 10.1016/j.cej.2019.04.213

