# Supplementary material for: Unraveling the functional instability of bacterial consortia in crude oil degradation via integrated co-occurrence networks
Source: Front Microbiol. 2023 Oct 12;14:1270916. doi: 10.3389/fmicb.2023.1270916 (PMC10602786; doi:10.3389/fmicb.2023.1270916)
Supplement: Supplementary file 1 [file Data_Sheet_1.pdf]

# Supplementary material

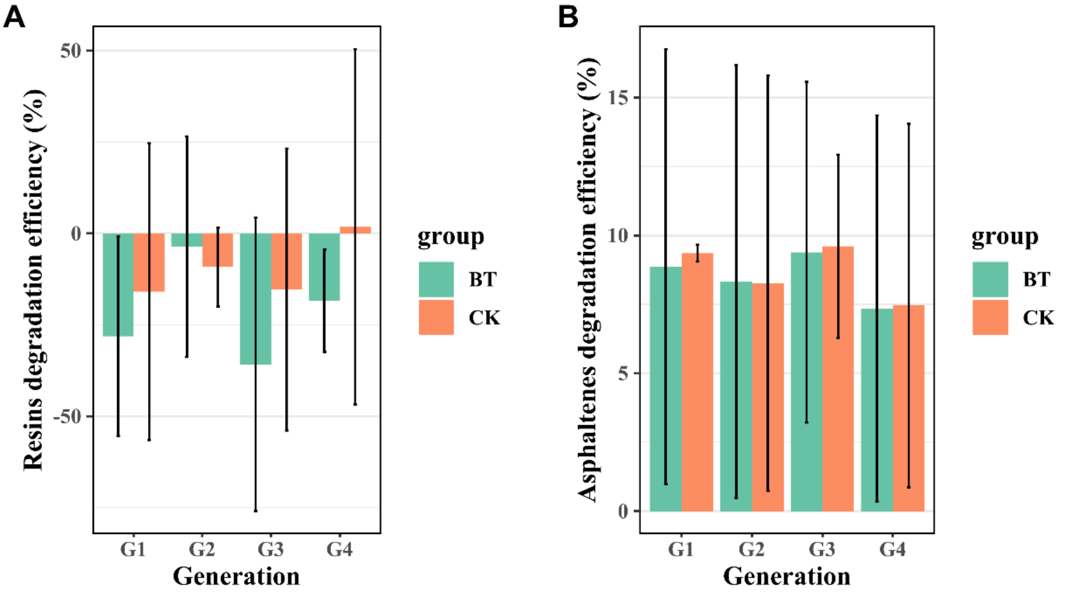


Fig. S1. Degradation efficiency of crude oil and its two fractions in four successive transfers: (A) of Resins degradation efficiency (B) Asphaltenes degradation efficiency.

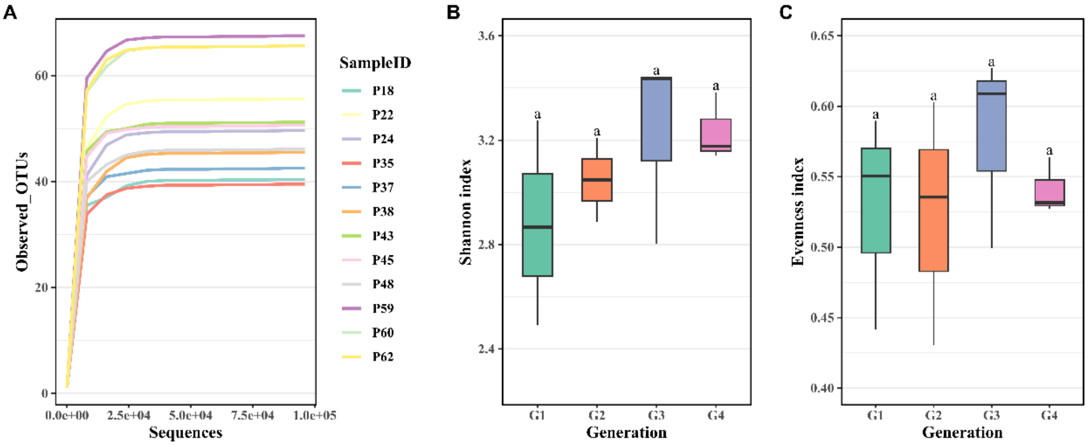

D

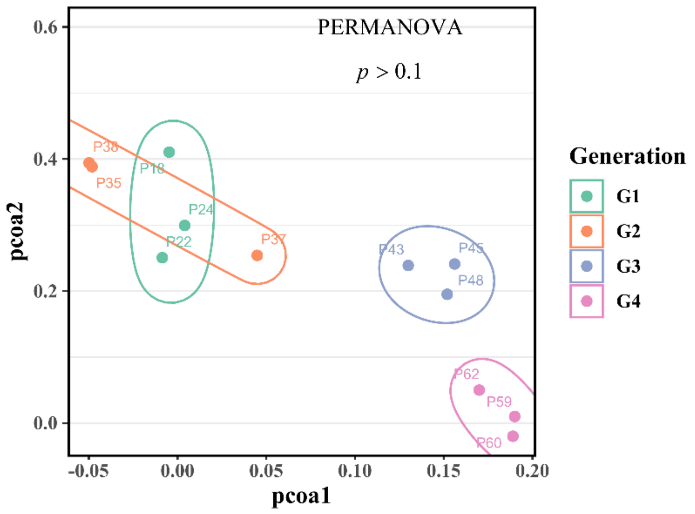


Fig. S2. Alpha diversity and beta diversity results of bacterial consortium. (A) Rarefaction curves of all samples. (B) Shannon index of bacterial consortium. (C) Evenness index of bacterial consortium. (D) PCoA of bacterial consortium. Within each column, means with the same letters are not significantly different at $p < 0.05$.

Table S1

key–score of the top 10 genera and relative abundance along successive transfer cultures.

| key–score *descending order* | G1 | G2 | G3 | G4 |
|---|---|---|---|---|
| 1 | *Pseudomonas*<br>0.04, 18.00% | *Arthrobacter*<br>0.0435, 0.21% | *Taonella*<br>0.0349, 0.03% | *Lysobacter*<br>0.046, 1.77% |
| 2 | *Dietzia*<br>0.0362, 20.00% | *Luteimonas*<br>0.0429, 0.09% | *Ralstonia*<br>0.0348, 0.12% | *Tepidiphilus*<br>0.0439, 0.01% |
| 3 | *Microbacterium*<br>0.0355, 4.60% | *Pseudomonas*<br>0.0389, 34.72% | *Lysinibacillus*<br>0.03479, 0.01% | *Novosphingobium*<br>0.0402, 6.89% |
| 4 | *Acinetobacter*<br>0.0352, 3.60% | *Cellulosimicrobium*<br>0.0382, 0.02% | *Pusillimonas*<br>0.03479, 0.02% | *Pseudomonas*<br>0.0399, 7.93% |
| 5 | *Sporosarcina*<br>0.035, 0.24% | *Dyella*<br>0.0378, 0.06% | *Longimicrobiaceae*<br>0.03479, 0.08% | *JG30.KF.CM45*<br>0.0376, 0.09% |
| 6 | *JG30.KF.CM45*<br>0.0348, 0.04% | *Microbacterium*<br>0.0361, 4.81% | *Georgenia*<br>0.03479, 0.01% | *Psychrobacter*<br>0.0372, 0.25% |
| 7 | *Tistlia*<br>0.0347, 0.06% | *Caulobacter*<br>0.0360, 0.13% | *Arthrobacter*<br>0.03479, 0.00% | *Brevibacillus*<br>0.0367, 0.01% |
| 8 | *Cellulosimicrobium*<br>0.0345, 0.03% | *Xanthomonas*<br>0.0355, 0.03% | *Pseudomonas*<br>0.0345, 7.68% | *Stenotrophomonas*<br>0.0365, 0.01% |
| 9 | *Microvirga*<br>0.0339, 0.00% | *Paenibacillus*<br>0.03522, 13.52% | *Luteibacter*<br>0.0344, 0.81% | *Rhodanobacter*<br>0.0364, 0.07% |
| 10 | *Luteimonas*<br>0.0338, 0.69% | *Pedobacter*<br>0.03521, 0.17% | *Agromyces*<br>0.0341, 0.28% | *Sporosarcina*<br>0.0363, 6.97% |
